# Hospital capacity for patient engagement in planning and improving health services: a cross-sectional survey

**DOI:** 10.1186/s12913-021-06174-0

**Published:** 2021-02-25

**Authors:** Anna R. Gagliardi, Juan Pablo Diaz Martinez, G. Ross Baker, Lesley Moody, Kerseri Scane, Robin Urquhart, Walter P. Wodchis

**Affiliations:** 1 Toronto General Hospital Research Institute, University Health Network, 200 Elizabeth Street, 13EN-228, Toronto, M5G2C4 Canada; 2 Biostatistics Research Unit, University Health Network, Toronto, Canada; 3 Institute of Health Policy, Management and Evaluation, University of Toronto, Toronto, Canada; 4 Princess Margaret Cancer Centre, University Health Network, Toronto, Canada; 5 Patient Partnerships, University Health Network, Toronto, Canada; 6 Department of Community Health and Epidemiology, Dalhousie University, Halifax, Nova Scotia Canada

**Keywords:** Patient engagement, Patient-centred care, Hospitals, Organizational capacity, Questionnaire

## Abstract

**Background:**

Patient engagement (PE) in planning or improving hospital facilities or services is one approach for improving healthcare delivery and outcomes. To provide evidence on hospital capacity needed to support PE, we described the attributes of hospital PE capacity associated with clinical quality measures.

**Methods:**

We conducted a cross-sectional survey of general and specialty hospitals based on the Measuring Organizational Readiness for Patient Engagement framework. We derived a PE capacity index measure, and with Multiple Correspondence Analysis, assessed the association of PE capacity with hospital type, and rates of hand-washing, *C. difficile* infection rates and 30-day readmission.

**Results:**

Respondents (91, 66.4%) included general: < 100 beds (48.4%), 100+ beds (27.5%), teaching hospitals (11.0%) and specialty (13.2%) hospitals. Most featured PE in multiple clinical and corporate departments. Most employed PE in a range of Planning (design/improve facilities 94.5%, develop strategic plans 87.9%), Evaluation/Quality Improvement (accreditation 91.2%, develop QI plans 90.1%) and Service Delivery activities (develop information/communication aids 92.3%). Hospitals enabled PE with multiple supports (median 12, range 0 to 25), most often: 76.9% strategic plan recognizes PE, 74.7% patient/family advisory council, and 69.2% pool of patient volunteers; and least often: 30.0% PE staff, 26.4% PE funding and 16.5% patient reimbursement or 3.3% compensation. Hospitals employed a range of less (inform, consult) and more (involve, partner) active modes of engagement. Two variables accounted for 29.6% of variance in hospital PE capacity index measure data: number of departments featuring PE and greater use of active engagement modes. PE capacity was not associated with general hospital type or clinical quality measures.

**Conclusions:**

Hospitals with fewer resources can establish favourable PE conditions by deploying PE widely and actively engaging patients. Healthcare policy-makers, hospital executives and PE managers can use these findings to allocate PE resources. Future research should explore how PE modes and methods impact clinical outcomes.

**Supplementary Information:**

The online version contains supplementary material available at 10.1186/s12913-021-06174-0.

## Introduction

Hospitals, which provide a considerable proportion of any country’s healthcare services, strive to improve patient experiences and outcomes. Hospitals provide inpatient, outpatient and emergency services, and account for the largest share of health spending in many countries, contributing to rising health expenditures worldwide, particularly in middle- and higher-income countries [1]. Across 27 countries, a median of 10% of hospital patients experienced at least one adverse event, largely stemming from surgical procedures, medication/fluids, and healthcare-associated infections, with a median of 51.2% considered preventable [2]. Hospital experiences, including adverse events, can negatively influence patient ratings of hospital, physician and nursing care [3]. The proportion of patients who gave high overall ratings of their hospital ranged from 35 to 60% across 13 countries [4]. In Canada, a survey of over 90,000 Canadians discharged from more than 300 acute care hospitals in 5 provinces between 2015 and 2018, 54% said their medication was always well-explained, 56% felt their care was always well-coordinated by hospital staff, and 66% felt completely informed of their condition, treatment and medication upon discharge [5]. Clearly, there is a need to further improve hospital patient experiences and care.

One approach for optimizing the quality and safety of hospital care is to engage patients (and/or family/care partners) in planning, evaluating and improving services for the benefit of all patients. In this context, patient engagement (PE) is defined as involving patients or their representatives in single (e.g. questionnaire, focus group) or ongoing (e.g. project team, standing committee) activities to plan, deploy, evaluate or improve hospital facilities, programs and services [6]. A scoping review of 10 studies published from 2006 to 2016 showed that evidence on the impact of PE in hospitals is still accumulating [7]. However, a systematic review of 48 studies published from 1990 to 2016 on PE in other settings showed that PE resulted in a range of benefits: 35 studies reported enhanced care or service delivery (e.g. extended hours, implementation of a care advocate, creation of new services, improved access to services), 15 studies reported developing policy or planning documents (e.g. clinical care models, strategic plans), 11 studies reported developing educational tools (e.g. patient information packages) and 5 studies reported enhancing governance processes (e.g. policy audit, organizational culture change) [8]. However, lack of organizational capacity to foster and support PE can lead to token PE with little or no service improvement [9, 10]. Moreover, there is little evidence-based guidance on approaches, models, infrastructure or methods that optimize hospital PE [11].

The goal of this research was to understand how to support and implement hospital PE. To address this goal, we asked: What PE activities do hospitals undertake and what is the mode of patient engagement in those activities; how does organizational capacity support hospital PE; and what is the association of hospital PE capacity with hospital type and clinical quality measures?

## Methods

### Approach

We used a cross-sectional survey design and complied with criteria for reporting survey research [12]. This approach explicitly describes participants’ views and experiences; it does not involve psychometric testing, and does not employ or generate theory as would an analytic or explanatory survey. The University Health Network Research Ethics Board approved the study (REB #18–5307). The research team, including four health services researchers, three patient research partners, two with PE experience at two different Ontario hospitals, two patient engagement managers, a biostatistician, and representatives of the Ontario Ministry of Health, Ontario Hospital Association, and Canadian healthcare accreditation agency contributed to research design and planning, survey development, data analysis, and interpretation of the findings.

### Sampling and recruitment

We used convenience sampling to recruit hospital corporations in Ontario, Canada, all of which are publicly funded. General (< 100 beds, 100+ beds, academic) and specialty (pediatric, cancer, rehabilitation, chronic/long-term care, psychiatric) hospitals were eligible, for a total of 137 hospitals based on the Ontario Ministry of Health web site on April 1, 2018. On our behalf, the Ontario Hospital Association (OHA) contacted hospitals on May 10, 2018 via an email group that included PE directors and managers with a link to the online survey, and sent two reminder emails (June 7 and June 18, 2018). To assist with recruitment, Cancer Care Ontario sent an email to PE managers at 14 sites (June 11, 2018) as did the Patient Engagement Community of Practice manager to 70+ hospitals (May 29 and June 7, 2018). To enhance response rate, we also acquired contact information (email, telephone number) for PE directors or managers at hospitals contacted by the OHA who had not yet responded from hospital web sites or by calling the hospital, and placed reminder phone calls on July 18, August 7 and August 22, 2018. We closed the survey on September 30, 2018.

### Data collection

Survey content reflected three concepts: organizational capacity for PE, activities in which patients or their representatives were engaged, and mode of engagement. Organizational capacity for PE reflected Oostendorp et al.’s Measuring Organizational Readiness for Patient Engagement (MORE) framework, which was developed via literature search, analysis of pre-existing tools, and a two-round online Delphi survey completed by 131 healthcare managers, policy makers, clinicians, patients, and researchers from 16 countries in round one, and 72 in round two [13]. MORE includes 21 items describing an organization’s ability to support PE organized as tasks, resources and situational factors. We derived PE activities from prior reviews and input from the research team, and categorized PE activities as Planning, Service Delivery, or Evaluation/Quality Improvement [7, 8]. We initially described three modes of engagement based on Carman et al.’s Framework of Patient Engagement: inform (provide patients with information about decisions via newsletter, town hall meeting, etc.), consult (solicit input or feedback via interview, focus group, or questionnaire) and partner (patients have the power to make decisions) [14]. Based on thorough review by the research team, we add one additional mode: involve (discussion via committees or project groups). The final survey included six sections: length of time that PE has been deployed (years), clinical services or corporate departments featuring PE (select all that apply), organizational support for PE (21 MORE items plus part-time/full-time PE manager, part-time/full-time PE staff, patient and family advisory committee, and reimbursement or compensation for patients for a total of 28 items, select all that apply), and mode of engagement (inform, consult, involve, partner, select all that apply) for three categories of activities: Planning (13 items, select all that apply), Service Delivery (14 items, select all that apply), and Evaluation/Quality Improvement (17 items, select all that apply). We administered the survey online with an introduction, purpose statement and instructions, noting that completion of the survey constituted informed consent.

### Data analysis

Although the survey instructed respondents to ensure that only one individual complete the survey on behalf of the organization, we reviewed all returned surveys for duplicates, and used the most recent or complete response from the PE director or manager. We used summary statistics to describe response rate, length of time that PE was deployed, number of clinical services or corporate departments featuring PE (not adjusted by type of hospital), organizational capacity for PE, and Planning, Service Delivery and Evaluation/Quality Improvement PE activities at hospitals overall and by hospital type including general (< 100 beds, 100+ beds, teaching) and specialty (pediatric, cancer, rehabilitation, chronic, psychiatric), which were grouped together given few respondents.

To further assess if hospital type was associated with organizational capacity for PE, we created an index measure of PE capacity. For this analysis we included only general hospitals (< 100 beds, 100+ beds, teaching) for a homogeneous sample and because so few specialty hospitals responded. The index measure was comprised of select factors identified in prior research [7, 8, 13] and consensus among the research team on factors where a higher value was likely to be associated with greater PE capacity. The initial model included the following factors: length of time involved with PE (years), number of clinical or corporate departments featuring PE, number of organizational supports for PE, and proportion of activities employing involve and partner (more engaged) compared with inform and consult (less engaged) modes of engagement. While both may be appropriate and needed, Bombard et al. showed that more engagement appeared to enhance care processes, service delivery and governance, whereas less engagement was more likely to generate discrete products such as patient educational material [8]. We used Multiple Correspondence Analysis (MCA), an approach that is increasingly used to examine complex data that shows if and how variables are related, and particularly useful for analyzing categorical survey data [15]. Similar to other multivariate methods, this approach assessed patterns in the date to establish which factors in the model contributed to PE capacity, and categorized hospitals as higher or lower capacity for PE. The benefit of MCA is that it groups respondents with similar profiles based on their answers and graphically displays the association between variable categories in a dimensional space for interpretation.

We also used the MCA analysis to assess the association between hospital capacity for PE and three quality measures, chosen on the premise that higher PE capacity would influence hospital outcomes, and because data were publicly-available and complete for the majority of hospitals: percent of opportunities where providers should and did wash their hands before patient contact (<organization name> 2017/18), *C. difficile* infection rate per 1000 inpatient days (<organization name> October 1 to December 31, 2018), and percent of patients readmitted to hospital within 30 days of discharge (<organization name> 2017/18).

## Results

### Respondents

**Fig. 1:**
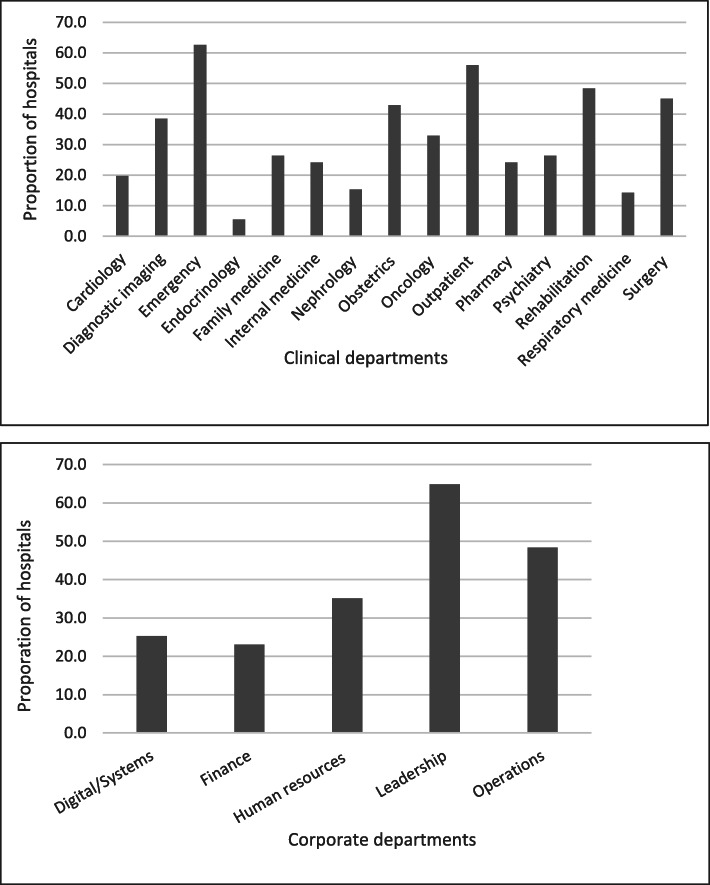
Proportion of hospitals featuring PE in clinical and corporate departments. Bar chart showing proportion of hospitals overall with PE activity in various departments

Directors or managers of PE from 91 (66.4%) hospitals responded (Table 1). The majority were general hospitals: < 100 beds (44, 48.4%), 100+ beds (25, 27.5%) and teaching hospitals (10, 11.0%). Specialty hospitals (12, 13.2%) included 6 rehabilitation, 3 psychiatric, 2 pediatric and 1 long-term care. The largest proportion of hospitals had engaged patients for 3 to 5 years (38, 41.8%) and 82 (90.1%) had engaged patients for at least 1 year. By hospital type, teaching and specialty hospitals were more likely to have more years of PE experience compared with hospitals of < 100 beds or 100+ beds (*p* = 0.007). The 91 hospitals featured PE in multiple clinical and corporate departments (Fig. 1). A higher proportion of teaching hospitals featured PE in the majority of clinical and corporate departments (*p* < 0.001).

**Table 1:** Length of time involved in PE

Length of time involved in PE (years)	Overall n (% of 91)	Hospital type n (%)				
		< 100 beds (*n* = 44)	≥100 beds (*n* = 25)	Teaching (*n* = 10)	Other (*n* = 12)	*p*-value
1 to 2	20 (20.9)	14 (31.8)	2 (8.0)	2 (20.0)	1 (8.3)	0.007
3 to 5	38 (41.8)	16 (36.4)	16 (64.0)	3 (30.0)	3 (25.0)	
6 or more	25 (27.5)	7 (15.9)	6 (24.0)	4 (40.0)	8 (66.7)	
< 1 year	7 (7.7)	6 (13.6)	1 (4.0)	0 (0.0)	0 (0.0)	
Not sure	2 (2.2)	1 (2.3)	0 (0.0)	1 (10.0)	0 (0.0)

### Support for PE

Many hospitals featured multiple (median 12, range 0 to 25) resources or processes that support PE (Table 2). While the median number of PE supports varied by hospital type, the association of support for PE with hospital type was inconsistent, and hospitals with a higher number of PE resources/processes were represented in each type: < 100 beds (median 7, range 0 to 23), 100+ beds (median 14, range 6 to 24), teaching (median 16, range 7 to 22), and specialty (median 17, range 3 to 25). Most hospitals had a strategic plan that included PE (76.9%), at least one patient and family advisory council (74.7%) and a pool of PE volunteers (69.2%). Fewer hospitals featured other resources or processes to support PE: full-time (30.8%) or part-time (29.7%) staff dedicated to PE, operational funding dedicated to PE (26.4%), PE training for clinicians/staff (31.9%) or joint patient-clinician/staff PE training (12.1%), reimbursement to patients for costs incurred in PE (16.5%) or compensation for patient time in PE (3.3%).

**Table 2:** Organizational capacity for PE

Organization support	Overall n (% of 91)	Hospital type n (%)				
		< 100 beds (*n* = 44)	≥100 beds (*n* = 25)	Teaching (*n* = 10)	Specialty (*n* = 12)	*p*-value
**Resources**						
Hospital strategic plan includes PE	70 (76.9)	28 (63.6)	22 (88.0)	8 (80.0)	12 (100.0)	0.021
Executive responsible for PE on a part time basis	55 (60.4)	25 (56.8)	17 (68.0)	8 (80.0)	5 (41.7)	NS
Board Member/Committee of the Board for PE	52 (57.1)	23 (52.3)	14 (56.0)	8 (80.0)	7 (58.3)	NS
Manager responsible for PE on a part time basis	42 (46.2)	16 (36.4)	15 (60.0)	5 (50.0)	6 (50.0)	NS
Policy specific to PE	34 (37.4)	15 (34.1)	11 (44.0)	4 (40.0)	4 (33.3)	NS
Full-time staff working for PE manager on PE	28 (30.8)	8 (18.2)	8 (32.0)	5 (50.0)	7 (58.3)	0.001
Part-time staff working for PE manager on PE	27 (29.7)	12 (27.3)	8 (32.0)	5 (50.0)	2 (16.7)	NS
Operational funding dedicated to PE activities	24 (26.4)	5 (11.4)	7 (28.0)	4 (40.0)	8 (66.7)	0.001
Reimbursement for costs incurred for PE activity	15 (16.5)	1 (2.3)	2 (8.0)	5 (50.0)	7 (58.3)	< 0.001
Manager responsible for PE on a full time basis	10 (11.0)	0 (0.0)	5 (20.0)	3 (30.0)	2 (16.7)	0.005
Executive responsible for PE on a full time basis	6 (6.6)	2 (4.5)	2 (8.0)	0 (0.0)	2 (16.7)	NS
Compensation for patient time on PE activity	3 (3.3)	1 (2.3)	1 (4.0)	1 (10.0)	0 (0.0)	NS
**Processes**						
At least 1 PFAC for clinical activities	68 (74.7)	28 (63.6)	23 (92.0)	8 (80.0)	9 (75.0)	NS
Pool of patient PE volunteers	64 (70.3)	25 (56.8)	20 (80.0)	10 (100.0)	9 (75.0)	NS
At least 1 PFAC for corporate activities	60 (65.9)	22 (50.0)	21 (84.0)	8 (80.0)	9 (75.0)	0.013
Senior executives promote or incentivize PE	59 (64.8)	22 (50.0)	22 (88.0)	6 (60.0)	9 (75.0)	0.013
PE is recognized/disseminated/celebrated	56 (61.5)	19 (43.2)	21 (84.0)	7 (70.0)	9 (75.0)	0.005
Staff liaison for patients involved in PE	56 (61.5)	21 (47.7)	19 (76.0)	8 (80.0)	8 (66.7)	NS
Chair/Co-Chair of PFAC is a patient	55 (60.4)	20 (45.5)	18 (72.0)	8 (80.0)	9 (75.0)	0.042
PE training for patients	46 (50.5)	13 (29.5)	16 (64.0)	8 (80.0)	9 (75.0)	0.001
Debriefing of patients after PE	38 (41.8)	9 (20.5)	16 (64.0)	6 (60.0)	7 (58.3)	0.001
Regular formal evaluation of PE processes/impact	34 (37.4)	11 (25.0)	12 (48.0)	4 (40.0)	7 (58.3)	NS
Patient involvement in evaluation of PE	34 (37.4)	9 (20.5)	12 (48.0)	5 (50.0)	8 (66.7)	0.009
Ongoing training for patients after onboarding	34 (37.4)	8 (18.2)	14 (56.0)	6 (60.0)	6 (50.0)	0.003
Patient liaison for patients involved in PE	32 (35.2)	7 (15.9)	10 (40.0)	6 (60.0)	9 (75.0)	< 0.001
PE training for clinicians/staff	29 (31.9)	7 (15.9)	8 (32.0)	7 (70.0)	7 (58.3)	0.001
Debriefing of clinicians/staff after PE	28 (30.8)	7 (15.9)	8 (32.0)	6 (60.0)	7 (58.3)	0.005
Joint patient-clinician/staff PE training	11 (12.1)	2 (4.5)	3 (12.0)	2 (20.0)	4 (33.3)	0.045

*PFAC* patient and family advisory committee, *NS* not significant

### PE activities

Hospitals employed PE in a wide range of Planning, Evaluation/Quality Improvement and Service Delivery activities, and employed one or more modes of engagement for these activities. The association between hospital type and engagement mode was inconsistent for all three types of activities (Additional files 1, 2 and 3).

Most hospitals employed PE in Planning activities to: design or improve hospital facilities (94.5%), develop strategic or operating plans (87.9%), develop policies or standards (86.8%) or establish clinical service priorities (80.2%) (Table 3). PE was least employed in interviewing/hiring healthcare staff or professionals (47.3%), establishing healthcare staff/professional competency frameworks (38.5%) or reviewing healthcare staff/professional performance (20.9%). Hospitals employed all modes of engagement in these activities, most often using consultation and less often using partnering apart from two exceptions: the inform mode was most often used when planning or reviewing hospital governance, and partner mode was most often used when interviewing or hiring healthcare staff or professionals.

**Table 3:** PE in hospital planning activities

Activity	Overall n (% 91)	Engagement mode			
		Inform	Consult	Involve	Partner
Design or improve hospital facilities	86 (94.5)	16 (17.6)	51 (56.0)	40 (44.0)	18 (19.8)
Develop strategic or operating plans	80 (87.9)	22 (24.2)	39 (42.9)	40 (44.0)	18 (19.8)
Develop policies or standards	79 (86.8)	22 (24.2)	37 (40.7)	35 (38.5)	15 (16.5)
Establish clinical service priorities	73 (80.2)	19 (20.9)	45 (49.5)	25 (27.5)	9 (9.9)
Develop plans to implement policies/strategies	70 (76.9)	19 (20.9)	39 (42.9)	24 (26.4)	10 (11.0)
Design ancillary services	68 (74.7)	22 (24.2)	33 (36.3)	30 (33.0)	12 (13.2)
Collect data to inform policies/plans	64 (70.3)	19 (20.9)	38 (41.8)	23 (25.3)	10 (11.0)
Plan/review hospital governance	58 (63.7)	33 (36.3)	22 (24.2)	13 (14.3)	3 (3.3)
Design information or e-health systems	57 (62.6)	22 (24.2)	26 (28.6)	21 (23.1)	6 (6.6)
Develop healthcare worker training curricula	47 (51.6)	14 (15.4)	25 (27.5)	15 (16.5)	8 (8.8)
Interview or hire healthcare workers	43 (47.3)	9 (9.9)	8 (8.8)	19 (20.9)	22 (24.2)
Establish healthcare worker competencies	35 (38.5)	11 (12.1)	12 (13.2)	12 (13.2)	4 (4.4)
Review healthcare worker performance	19 (20.9)	3 (3.3)	5 (5.5)	2 (2.2)	3 (3.3)

Most hospitals employed PE in Evaluation/Quality Improvement activities to: plan for or participate in accreditation (91.2%), develop strategic or operating plans for quality and safety (90.1%) or develop quality criteria/indicators (84.6%) (Table 4). PE was least employed to: train healthcare staff/professionals to implement new or improved services (29.7%), plan or conduct healthcare staff/professional interviews or focus groups to inform audit (25.3%) or plan or conduct simulated patient visits (18.7%). Hospitals employed all modes of engagement in these activities, most often with consult or involve engagement modes.

**Table 4:** PE in hospital evaluation and quality improvement activities

Activity	Overall n (% 91)	Engagement mode			
		Inform	Consult	Involve	Partner
Plan for or participate in accreditation	83 (91.2)	26 (28.6)	39 (42.9)	40 (44.0)	22 (24.2)
Develop strategic or operating plans for quality and safety	82 (90.1)	20 (22.0)	45 (49.5)	38 (41.8)	16 (17.6)
Develop quality criteria/indicators	77 (84.6)	16 (17.6)	34 (37.4)	34 (37.4)	15 (16.5)
Design or plan specific quality improvement projects	69 (75.8)	20 (22.0)	30 (33.0)	34 (37.4)	17 (18.7)
Review audit data	55 (60.4)	21 (23.1)	18 (19.8)	24 (26.4)	8 (8.8)
Conduct or execute quality improvement projects	54 (59.3)	15 (16.5)	23 (25.3)	23 (25.3)	14 (15.4)
Implement changes emerging from quality improvement projects	53 (58.2)	21 (23.1)	20 (22.0)	14 (15.4)	12 (13.2)
Develop patient data collection instruments to inform audit	46 (50.5)	11 (12.1)	20 (22.0)	19 (20.9)	10 (11.0)
Design improved clinical services based on audit findings	45 (49.5)	12 (13.2)	18 (19.8)	20 (22.0)	9 (9.9)
Plan or conduct patient interviews/focus groups to inform audit	42 (46.2)	10 (11.0)	19 (20.9)	18 (19.8)	8 (8.8)
Audit a clinical or hospital service	41 (45.1)	11 (12.1)	11 (12.1)	22 (24.2)	9 (9.9)
Plan or conduct executive walkabouts	37 (40.7)	13 (14.3)	9 (9.9)	15 (16.5)	7 (7.7)
Develop the format/content of audit reports	30 (33.0)	6 (6.6)	10 (11.0)	11 (12.1)	4 (4.4)
Design healthcare staff/professional data collection instruments to inform audit	28 (30.8)	8 (8.8)	6 (6.6)	10 (11.0)	3 (3.3)
Train healthcare staff/professionals to implement new or improve existing services	27 (29.7)	7 (7.7)	9 (9.9)	9 (9.9)	7 (7.7)
Plan or conduct healthcare staff/professional interviews or focus groups to inform audit	23 (25.3)	5 (5.5)	6 (6.6)	7 (7.7)	4 (4.4)
Plan or conduct simulated patient visits	17 (18.7)	4 (4.4)	4 (4.4)	6 (6.6)	1 (1.1)

Fewer hospitals engaged patients in Service Delivery activities (Table 5). Most employed PE to develop information or communication aids (92.3%), or develop patient satisfaction or experience data collection instruments (83.5%). PE was least employed in delivering psycho-social or emotional support programs (31.9%), or in designing training programs for patient navigators (27.5%). Hospitals employed all modes of engagement in these activities, most often using consultation. Notably, more hospitals used the partner mode over other modes to: deliver presentations about PE activities or impact to internal or external audiences, and to provide PE orientation or training to healthcare staff, professionals or executives.

**Table 5:** PE in hospital service delivery activities

Activity	Overall n (% 91)	Engagement mode			
		Inform	Consult	Involve	Partner
Develop information or communication aids	84 (92.3)	20 (22.0)	29 (31.9)	42 (46.2)	32 (35.2)
Develop patient satisfaction or experience instruments	76 (83.5)	22 (24.2)	34 (37.4)	38 (41.8)	23 (25.3)
Design clinics and rooms	67 (73.6)	19 (20.9)	33 (36.3)	32 (35.2)	10 (11.0)
Provide PE orientation/training to new PFAC members	56 (61.5)	12 (13.2)	22 (24.2)	18 (19.8)	23 (25.3)
Design patient education programs	54 (59.3)	12 (13.2)	28 (30.8)	24 (26.4)	16 (17.6)
Deliver external presentations about PE activities/impact	45 (49.5)	8 (8.8)	12 (13.2)	17 (18.7)	23 (25.3)
Design care pathways	44 (48.4)	12 (13.2)	27 (29.7)	15 (16.5)	9 (9.9)
Deliver internal presentations about PE activities/impact	44 (48.4)	10 (11.0)	16 (17.6)	16 (17.6)	20 (22.0)
Provide PE orientation or training to healthcare staff, professionals or executives	38 (41.8)	7 (7.7)	14 (15.4)	11 (12.1)	16 (17.6)
Deliver one-on-one or group patient education	34 (37.4)	10 (11.0)	13 (14.3)	8 (8.8)	12 (13.2)
Design psycho-social/emotional support programs	34 (37.4)	10 (11.0)	14 (15.4)	10 (11.0)	11 (12.1)
Function as patient navigators	33 (36.3)	7 (7.7)	11 (12.1)	10 (11.0)	7 (7.7)
Deliver psycho-social/emotional support programs	29 (31.9)	6 (6.6)	9 (9.9)	7 (7.7)	6 (6.6)
Design patient navigator training programs	25 (27.5)	4 (4.4)	9 (9.9)	5 (5.5)	4 (4.4)

*PFAC* patient & family advisory committee

### Organizational capacity for PE and hospital type

**Fig. 2:**
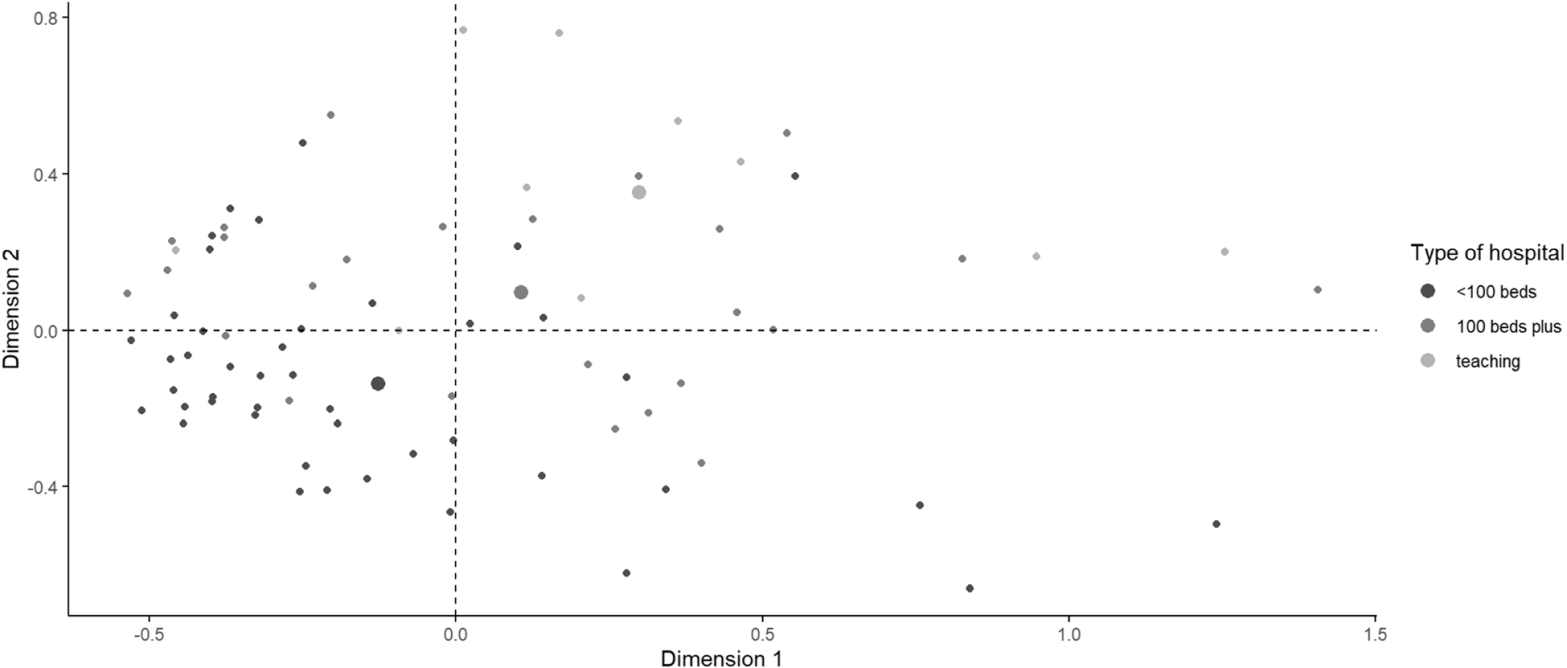
Organizational capacity for PE by hospital type. Multiple correspondence analysis quadrant plot showing hospital PE capacity

Two variables accounted for 29.6% of variance in data for the index measure of hospital PE capacity: number of departments featuring PE and higher proportion of more active engagement modes. Figure 2 displays the association between hospital capacity for PE and general hospital type. The dots represent hospitals that clustered due to common associations between variables; the further they are from the origin, the stronger the association between variables. Hospitals clustered in the two right quadrants is indicative of greater capacity for PE, particularly those in the upper right quadrant. Notably, general hospitals of all types (< 100 beds, 100+ beds, teaching hospitals) are represented among those in the two right quadrants as having higher PE capacity.

### Organizational capacity for PE and quality measures

Additional file 4 shows the association of hospital capacity for PE and pre-procedure hand-washing, *C. difficile* infection rates and 30-day readmission rates. Hospitals clustered on both sides of the X axis, with few hospitals to the right of the X axis representing favourable values for these quality measures, revealing an inconsistent association of organizational capacity for PE with the chosen measures.

## Discussion

We aimed to describe hospital PE activity and mode of PE in those activities; the organizational capacity of hospitals for PE; and the association of hospital PE capacity with hospital type and clinical quality measures. Regarding the first objective, findings revealed the wide-spread adoption of PE by hospitals. Hospitals of all types featured PE in multiple clinical/corporate departments; supported PE with multiple resources and/or processes; used PE for a wide range of Planning, Evaluation and Quality Improvement and Service Delivery activities; and engaged patients using a variety of modes including those requiring lesser (inform, consult) and greater (involve, partner) engagement. Our findings addressed the second objective by using an index measure of hospital PE capacity that distinguished general hospitals of each type (< 100 beds, 100+ beds, teaching) as having high PE capacity, which was largely attributed to the number of clinical and/or corporate departments featuring PE, and the proportion of activities employing involve and partner modes of engagement. With respect to objective three, hospital PE capacity was inconsistently associated with hospital type, or pre-procedure hand-washing, *C. difficile* infection rates and 30-day readmission rates.

Little prior research described hospital PE, required resources or processes, or its impact; instead, focusing largely on barriers of identifying and involving patients and healthcare professionals [6, 7]. Malloggi et al. surveyed 213 healthcare workers in a French university hospital, revealing they had engaged patients in developing care pathways, patient education programs, and continuing education of healthcare professionals [16]. A survey of 1457 American acute care hospitals focused on practices to engage patients in their own care rather than planning or improvement activities that benefit other patients [17]. Several other studies were based on qualitative research designs and identified numerous barriers to PE in quality improvement activities [18–20]. Thus, our study is unique from prior research, having generated epidemiologic data describing hospital PE activity and capacity, and how modes of PE, PE capacity and hospital type may beassociated with PE and related outcomes.

The results suggest several implications for policy, practice and ongoing research. Given widespread need to improve the quality of hospital care [2–5], and global attention on PE as a means of doing so [6–8], these implications are broadly relevant. Healthcare policy-makers, hospital executives and PE managers may identify PE infrastructure or processes that hospitals lack to inform resource allocation decisions. For example, many hospitals lacked staff or operational funding dedicated to PE. Lack of organizational resources has been identified as a barrier to PE [7–10, 18–20]. Given that PE appears to be quite prevalent in most hospitals who responded to our survey, this means that PE may be an add-on activity in most hospitals. Via case-study, engagement-capable environments were characterized as enlisting and preparing patients, engaging staff to involve patients, and ensuring leadership support [21]. This study showed that PE capacity was characterized by PE across multiple departments and modes of greater involvement. However, PE activities without dedicated support, relying on healthcare staff and professionals already burdened with multiple portfolios or responsibilities, may not fully achieve an engagement-capable milieu, possibly leading to token PE and limited service improvement as reported in prior research [9, 10]. In our study, few hospitals compensated or reimbursed patients for their time and expenses incurred in PE activities. Paying patients is increasingly viewed as a standard and necessary practice that optimizes PE by reducing power imbalances, showing respect for vulnerability and willingness to share their lived experience, demonstrating organizational commitment to PE and value for patient perspectives, and eliminating barriers to participation and enhancing equity and diversity [22]. While this survey and its results may provide decision-makers with considerations on how to allocate resources for PE, further research is needed to understand which resources and processes are most crucial for PE and associated outcomes to guide more targeted allocation decisions.

Hospitals may employ a “mosaic” of engagement modes and methods, which alleviates the expectation that a few select patients can represent the voices of all patients, and that including many voices through different types of engagement allows for a more robust understanding of patient experiences and preferences [23–25]. Bombard et al. synthesized 48 studies of PE in the design, delivery and evaluation of health services [8]. Reported PE outcomes included discrete products (e.g. educational tools, policies) and enhanced care delivery (e.g. care processes or services). Engagement mode appeared to influence PE outcomes. Discrete outputs largely derived from lower-level engagement whereas care delivery was enhanced through higher-level engagement. Our findings support the distinction in mode of engagement proposed by Bombard et al. by showing that hospitals with greater organizational capacity for PE were characterized in part by mode of engagement (involve, partner) in which patients are more active. This finding may help PE managers match mode of engagement to specific initiatives or projects depending on desired outcomes. Still, further research is needed to generate deeper insight on how to optimize the range of modes and methods of PE so that desired outcomes reflect patient needs and preferences.

Our research builds on work by Oostendoorp et al., who compiled published research and expert consensus to generate the MORE scale, which includes 21 resources and processes reflecting an organization’s ability to implement PE. In our research, some non-teaching hospitals with fewer PE resources and processes than teaching hospitals emerged as having high organizational capacity for PE. This finding underscores the relative importance of PE pervasiveness (number of departments featuring PE) and more active engagement mode. Pervasiveness and active engagement may reflect an organizational culture and leadership that embrace and promote PE, key components of an “engagement-capable environment” [21]. The lesson here is that even small hospitals can overcome the lack of dedicated resources by prioritizing and embedding PE in all activities. While MORE represents a comprehensive list of ideal resources and processes that support PE, our findings suggest some may be more crucial than others. Thus, as noted, further research is needed to identify a core set of the most important infrastructural elements so that even lower-resource settings can lobby for and allocate resources to those essential approaches.

Further research is also needed in two areas: hospital support for PE and the impact of PE. In our study, hospital support for PE did not emerge as a predominant contributor to variability in PE capacity. However, it stands to reason that more support may enable greater activity in multiple departments with possibly more active modes of engagement. However, the role or mechanism of particular aspects of organizational support associated with PE capacity remains to be established. In our study, measures of clinical quality, chosen because data were publicly available, were not associated with PE capacity and may not be directly relevant to PE, though we cannot say with certainty why this was the case. Synthesis of published research showed that PE enhanced governance processes and service delivery, and generated policies, plans and educational tools, which hypothetically should improve patient-important outcomes such as healthcare experiences as well as clinical outcomes [8]. Prior research was largely conducted in primary care rather than hospital settings [7, 8]. Therefore, future research should identify patient-important and clinical outcomes associated with PE in hospital settings. Strengths of this research included use of rigorous survey methods that complied with reporting standards [12], multiple points of input and review by an interdisciplinary research team that included three patient research partners with hospital PE experience, and a good response rate relative to typical survey response rates [26]. We contributed to a knowledge gap on PE in hospital settings, examined a wide range of organizational factors and their influence on PE, and identified several issues that warrant ongoing research. Still, we must acknowledge some limitations. Our survey was based on MORE, which may not necessarily capture all PE resources and processes, but it was compiled using robust methods from published research and expert consensus, and represents the most comprehensive framework of PE supports given the lack of validated tools for assessing PE [27]. The data may have been influenced by self-report bias inherent in survey methods. The findings may not be relevant to specialty hospitals, as few responded and they were not included in statistical analyses of PE capacity, nor to hospitals in other types of health systems or countries. To address some of these limitations, we are interviewing hospital PE managers, patients and clinicians involved in PE activities to further explore how hospital capacity supports PE and related impacts. Based on those findings, we will establish expert consensus on essential elements of hospital PE capacity.

## Conclusions

Our survey of 91 general and specialty hospitals revealed that most hospitals engaged patients in planning, evaluation/quality improvement and service delivery using both more (involve, partner) and less (inform, consult) active modes of engagement across multiple clinical and corporate departments. We showed that hospital PE capacity is more dependent on PE pervasiveness (the number of clinical and/or corporate departments featuring PE) and active modes of engagement (involve, partner) regardless of general hospital type (< 100 beds, 100+ beds, teaching) compared with variables such as length of time involved in PE or organizational supports for PE. Thus, even lower-resource settings or hospitals with fewer resources can establish favourable PE conditions. This research addresses a gap in prior PE research by describing hospital PE activity and capacity, and how modes of PE, PE capacity and hospital type are associated with PE and related outcomes. Although this research was based in Canada, given widespread evidence of the need to improve hospital quality of care and attention to PE as a way of doing so, the findings are broadly relevant. Healthcare policy-makers, hospital executives and PE managers may refer to these findings to guide decisions about resources and processes to foster PE. Future research should explore how to optimize the range of modes and methods of PE so that desired outcomes reflect patient needs and preferences, establish a core set of the most important PE capacity elements, and identify clinical outcomes associated with PE capacity. Ultimately, by strengthening PE capacity, hospitals may better meet accreditation requirements, and the needs of the patients and families they serve.

## Supplementary Information

**Additional file 1.** PE planning by engagement mode hospital type. PE in planning activities by engagement mode and hospital type. Table showing summary statistics.

**Additional file 2.** Evaluation quality improvement by engagement mode hospital type. PE in evaluation/quality improvement activities by engagement mode and hospital type. Table showing summary statistics.

**Additional file 3.** PE service delivery by engagement mode hospital type. PE in service delivery activities by engagement mode and hospital type. Table showing summary statistics.

**Additional file 4.** PE capacity and quality measures. Association of organizational capacity for PE and quality measures. Multiple correspondence analysis grids.

## Data Availability

All data generated or analysed during this study are included in this published article and its supplementary information files.
