# Supplementary material for: Hospital capacity for patient engagement in planning and improving health services: a cross-sectional survey
Source: BMC Health Serv Res. 2021 Feb 25;21:179. doi: 10.1186/s12913-021-06174-0 (PMC7908767; doi:10.1186/s12913-021-06174-0)
Supplement: Supplementary file 1 — Additional file 1. PE planning by engagement mode hospital type. PE in planning activities by engagement mode and hospital type. Table showing summary statistics. [file 12913_2021_6174_MOESM1_ESM.docx]

**Hospital capacity for patient engagement in planning and improving health services: A cross-sectional survey**

Anna R Gagliardi*, Toronto General Hospital Research Institute, University Health Network, Toronto, Canada

Juan Pablo Diaz Martinez, Biostatistics Research Unit, University Health Network, Toronto, Canada

G. Ross Baker, Institute of Health Policy, Management and Evaluation, University of Toronto, Toronto, Canada

Lesley Moody, Princess Margaret Cancer Centre, University Health Network, Toronto, Canada

Kerseri Scane, Patient Partnerships, University Health Network, Toronto, Canada

Robin Urquhart, Department of Community Health and Epidemiology, Dalhousie University, Halifax, Nova Scotia

Walter Wodchis, Institute of Health Policy, Management and Evaluation, University of Toronto, Toronto, Canada

*Corresponding author:

anna.gagliardi@uhnresearch.ca

Toronto General Hospital, 200 Elizabeth Street, 13EN-228, Toronto, Canada, M5G2C4

Additional File 1. PE in planning activities by engagement mode and hospital type

| Activity | Overall  n (% of 91) | Mode of engagement | Overall by mode of engagement  n (% of 91) | Engagement mode by hospital type  n (%) | | | | |
| --- | --- | --- | --- | --- | --- | --- | --- | --- |
|  |  |  |  | <100 beds  (n=44) | 100+ beds (n=25) | Teaching  (n=10) | Specialty  (n=12) | p-value |
| Plan/review hospital governance | 58 (63.7) | Inform | 33 (36.3) | 16 (36.4) | 9 (36.0) | 1 (10.0) | 7 (58.3) | NS |
|  |  | Consult | 22 (24.2) | 10 (22.7) | 8 (32.0) | 2 (20.0) | 2 (16.7) | NS |
|  |  | Involve | 13 (14.3) | 3 (6.8) | 5 (20.0) | 2 (20.0) | 3 (25.0) | NS |
|  |  | Partner | 3 (3.3) | 0 (0.0) | 1 (4.0) | 0 (0.0) | 2 (16.7) | 0.035 |
| Design or improve hospital facilities | 86 (94.5) | Inform | 16 (17.6) | 5 (11.4) | 4 (16.0) | 1 (10.0) | 6 (50.0) | 0.016 |
|  |  | Consult | 51 (56.0) | 22 (50.0) | 13 (52.0) | 6 (60.0) | 10 (83.3) | NS |
|  |  | Involve | 40 (44.0) | 13 (29.5) | 13 (52.0) | 8 (80.0) | 6 (50.0) | 0.020 |
|  |  | Partner | 18 (19.8) | 5 (11.4) | 6 (24.0) | 3 (30.0) | 4 (33.3) | NS |
| Design ancillary services | 68 (74.7) | Inform | 22 (24.2) | 7 (15.9) | 6 (24.0) | 1 (10.0) | 8 (66.7) | 0.002 |
|  |  | Consult | 33 (36.3) | 14 (31.8) | 8 (32.0) | 4 (40.0) | 7 (58.3) | NS |
|  |  | Involve | 30 (33.0) | 10 (22.7) | 11 (44.0) | 6 (60.0) | 3 (25.0) | NS |
|  |  | Partner | 12 (13.2) | 4 (9.1) | 2 (8.0) | 2 (20.0) | 4 (33.3) | NS |
| Establish clinical service priorities | 73 (80.2) | Inform | 19 (20.9) | 5 (11.4) | 7 (28.0) | 1 (10.0) | 6 (50.0) | 0.018 |
|  |  | Consult | 45 (49.5) | 17 (38.6) | 11 (44.0) | 7 (70.0) | 10 (83.3) | 0.023 |
|  |  | Involve | 25 (27.5) | 8 (18.2) | 10 (40.0) | 5 (50.0) | 2 (16.7) | NS |
|  |  | Partner | 9 (9.9) | 1 (2.3) | 3 (12.0) | 3 (30.0) | 2 (16.7) | 0.043 |
| Develop strategic or operating plans | 80 (87.9) | Inform | 22 (24.2) | 9 (20.5) | 7 (28.0) | 0 (0.0) | 6 (50.0) | 0.044 |
|  |  | Consult | 39 (42.9) | 16 (36.4) | 9 (36.0) | 4 (40.0) | 10 (83.3) | 0.026 |
|  |  | Involve | 40 (44.0) | 13 (29.5) | 13 (52.0) | 9 (90.0) | 5 (41.7) | 0.005 |
|  |  | Partner | 18 (19.8) | 6 (13.6) | 7 (28.0) | 2 (20.0) | 3 (25.0) | NS |
| Develop policies or standards | 79 (86.8) | Inform | 22 (24.2) | 9 (20.5) | 5 (20.0) | 2 (20.0) | 6 (50.0) | NS |
|  |  | Consult | 37 (40.7) | 15 (34.1) | 12 (48.0) | 4 (40.0) | 6 (50.0) | NS |
|  |  | Involve | 35 (38.5) | 12 (27.3) | 10 (40.0) | 6 (60.0) | 7 (58.3) | NS |
|  |  | Partner | 15 (16.5) | 3 (6.8) | 5 (20.0) | 4 (40.0) | 3 (25.0) | 0.049 |
| Design information or e-health systems | 57 (62.6) | Inform | 22 (24.2) | 9 (20.5) | 6 (24.0) | 2 (20.0) | 5 (41.7) | NS |
|  |  | Consult | 26 (28.6) | 4 (9.1) | 11 (44.0) | 5 (50.0) | 6 (50.0) | 0.001 |
|  |  | Involve | 21 (23.1) | 2 (4.5) | 9 (36.0) | 7 (70.0) | 3 (25.0) | <0.001 |
|  |  | Partner | 6 (6.6) | 1 (2.3) | 1 (4.0) | 3 (30.0) | 1 (8.3) | 0.014 |
| Collect data from leaders or policy makers to inform policies/plans | 64 (70.3) | Inform | 19 (20.9) | 7 (15.9) | 6 (24.0) | 0 (0.0) | 6 (50.0) | 0.022 |
|  |  | Consult | 38 (41.8) | 16 (36.4) | 11 (44.0) | 4 (40.0) | 7 (58.3) | NS |
|  |  | Involve | 23 (25.3) | 8 (18.2) | 10 (40.0) | 4 (40.0) | 1 (8.3) | NS |
|  |  | Partner | 10 (11.0) | 2 (4.5) | 5 (20.0) | 1 (10.0) | 2 (16.7) | NS |
| Develop plans to implement policies and strategies | 70 (76.9) | Inform | 19 (20.9) | 7 (15.9) | 6 (24.0) | 1 (10.0) | 5 (41.7) | NS |
|  |  | Consult | 39 (42.9) | 18 (40.9) | 11 (44.0) | 4 (40.0) | 6 (50.0) | NS |
|  |  | Involve | 24 (26.4) | 7 (15.9) | 11 (44.0) | 4 (40.0) | 2 (16.7) | 0.046 |
|  |  | Partner | 10 (11.0) | 2 (4.5) | 4 (16.0) | 3 (30.0) | 1 (8.3) | NS |
| Develop training curricula for staff or healthcare professionals | 47 (51.6) | Inform | 14 (15.4) | 6 (13.6) | 6 (24.0) | 1 (10.0) | 1 (8.3) | NS |
|  |  | Consult | 25 (27.5) | 5 (11.4) | 11 (44.0) | 6 (60.0) | 3 (25.0) | 0.002 |
|  |  | Involve | 15 (16.5) | 4 (9.1) | 4 (16.0) | 4 (40.0) | 3 (25.0) | NS |
|  |  | Partner | 8 (8.8) | 0 (0.0) | 2 (8.0) | 3 (30.0) | 3 (25.0) | 0.003 |
| Establish hiring competencies for staff or healthcare professionals | 35 (38.5) | Inform | 11 (12.1) | 4 (9.1) | 3 (12.0) | 0 (0.0) | 4 (33.3) | NS |
|  |  | Consult | 12 (13.2) | 2 (4.5) | 2 (8.0) | 5 (50.0) | 3 (25.0) | 0.001 |
|  |  | Involve | 12 (13.2) | 3 (6.8) | 3 (12.0) | 3 (30.0) | 3 (25.0) | NS |
|  |  | Partner | 4 (4.4) | 1 (2.3) | 1 (4.0) | 1 (10.0) | 1 (8.3) | NS |
| Interview or hire healthcare professionals or staff | 43 (47.3) | Inform | 9 (9.9) | 4 (9.1) | 4 (16.0) | 0 (0.0) | 1 (8.3) | NS |
|  |  | Consult | 8 (8.8) | 1 (2.3) | 3 (12.0) | 1 (10.0) | 3 (25.0) | NS |
|  |  | Involve | 19 (20.9) | 4 (9.1) | 6 (24.0) | 4 (40.0) | 5 (41.7) | 0.027 |
|  |  | Partner | 22 (24.2) | 3 (6.8) | 8 (32.0) | 6 (60.0) | 5 (41.7) | 0.001 |
| Review staff or healthcare professional performance | 19 (20.9) | Inform | 3 (3.3) | 1 (2.3) | 0 (0.0) | 0 (0.0) | 2 (16.7) | 0.045 |
|  |  | Consult | 5 (5.5) | 1 (2.3) | 0 (0.0) | 2 (20.0) | 2 (16.7) | 0.026 |
|  |  | Involve | 2 (2.2) | 1 (2.3) | 0 (0.0) | 1 (10.0) | 0 (0.0) | NS |
|  |  | Partner | 3 (3.3) | 0 (0.0) | 1 (4.0) | 1 (10.0) | 1 (8.3) | NS |
