# Supplementary material for: Hospital capacity for patient engagement in planning and improving health services: a cross-sectional survey
Source: BMC Health Serv Res. 2021 Feb 25;21:179. doi: 10.1186/s12913-021-06174-0 (PMC7908767; doi:10.1186/s12913-021-06174-0)
Supplement: Supplementary file 2 — Additional file 2. Evaluation quality improvement by engagement mode hospital type. PE in evaluation/quality improvement activities by engagement mode and hospital type. Table showing summary statistics. [file 12913_2021_6174_MOESM2_ESM.docx]

**Hospital capacity for patient engagement in planning and improving health services: A cross-sectional survey**

Anna R Gagliardi*, Toronto General Hospital Research Institute, University Health Network, Toronto, Canada

Juan Pablo Diaz Martinez, Biostatistics Research Unit, University Health Network, Toronto, Canada

G. Ross Baker, Institute of Health Policy, Management and Evaluation, University of Toronto, Toronto, Canada

Lesley Moody, Princess Margaret Cancer Centre, University Health Network, Toronto, Canada

Kerseri Scane, Patient Partnerships, University Health Network, Toronto, Canada

Robin Urquhart, Department of Community Health and Epidemiology, Dalhousie University, Halifax, Nova Scotia

Walter Wodchis, Institute of Health Policy, Management and Evaluation, University of Toronto, Toronto, Canada

*Corresponding author:

anna.gagliardi@uhnresearch.ca

Toronto General Hospital, 200 Elizabeth Street, 13EN-228, Toronto, Canada, M5G2C4

Additional File 2. PE in evaluation/quality improvement activities by engagement mode and hospital type

| Activities | Overall  n (% of 91) | Engagement approach | Overall by mode of engagement  n (% of 91) | Engagement mode by hospital type  n (%) | | | | |
| --- | --- | --- | --- | --- | --- | --- | --- | --- |
|  |  |  |  | <100 beds  (n=44) | 100+ beds (n=25) | Teaching  (n=10) | Specialty  (n=12) | p-value |
| Develop strategic or operating plans for quality and safety | 82 (90.1) | Inform | 20 (22.0) | 8 (18.2) | 5 (20.0) | 2 (20.0) | 5 (41.7) | NS |
|  |  | Consult | 45 (49.5) | 18 (40.9) | 11 (44.0) | 6 (60.0) | 10 (83.3) | NS |
|  |  | Involve | 38 (41.8) | 17 (38.6) | 10 (40.0) | 7 (70.0) | 4 (33.3) | NS |
|  |  | Partner | 16 (17.6) | 3 (6.8) | 7 (28.0) | 3 (30.0) | 3 (25.0) | NS |
| Develop quality criteria/indicators | 77 (84.6) | Inform | 16 (17.6) | 7 (15.9) | 4 (16.0) | 1 (10.0) | 4 (33.3) | NS |
|  |  | Consult | 34 (37.4) | 17 (38.6) | 8 (32.0) | 3 (30.0) | 6 (50.0) | NS |
|  |  | Involve | 34 (37.4) | 15 (34.1) | 8 (32.0) | 6 (60.0) | 5 (41.7) | NS |
|  |  | Partner | 15 (16.5) | 3 (6.8) | 7 (28.0) | 2 (20.0) | 3 (25.0) | NS |
| Audit a clinical or hospital service | 41 (45.1) | Inform | 11 (12.1) | 5 (11.4) | 2 (8.0) | 0 (0.0) | 4 (33.3) | NS |
|  |  | Consult | 11 (12.1) | 4 (9.1) | 1 (4.0) | 2 (20.0) | 4 (33.3) | NS |
|  |  | Involve | 22 (24.2) | 6 (13.6) | 8 (32.0) | 4 (40.0) | 4 (33.3) | NS |
|  |  | Partner | 9 (9.9) | 1 (2.3) | 3 (12.0) | 2 (20.0) | 3 (25.0) | NS |
| Review audit data | 55 (60.4) | Inform | 21 (23.1) | 11 (25.0) | 5 (20.0) | 1 (10.0) | 4 (33.3) | NS |
|  |  | Consult | 18 (19.8) | 8 (18.2) | 4 (16.0) | 1 (10.0) | 5 (41.7) | NS |
|  |  | Involve | 24 (26.4) | 11 (25.0) | 7 (28.0) | 2 (20.0) | 4 (33.3) | NS |
|  |  | Partner | 8 (8.8) | 3 (6.8) | 1 (4.0) | 2 (20.0) | 2 (16.7) | NS |
| Develop the format/content of audit reports | 30 (33.0) | Inform | 6 (6.6) | 3 (6.8) | 1 (4.0) | 0 (0.0) | 2 (16.7) | NS |
|  |  | Consult | 10 (11.0) | 4 (9.1) | 1 (4.0) | 2 (20.0) | 3 (25.0) | NS |
|  |  | Involve | 11 (12.1) | 5 (11.4) | 2 (8.0) | 4 (40.0) | 0 (0.0) | 0.024 |
|  |  | Partner | 4 (4.4) | 1 (2.3) | 0 (0.0) | 2 (20.0) | 1 (8.3) | 0.049 |
| Design improved clinical services based on audit findings | 45 (49.5) | Inform | 12 (13.2) | 3 (6.8) | 3 (12.0) | 1 (10.0) | 5 (41.7) | 0.017 |
|  |  | Consult | 18 (19.8) | 5 (11.4) | 4 (16.0) | 4 (40.0) | 5 (41.7) | 0.039 |
|  |  | Involve | 20 (22.0) | 7 (15.9) | 6 (24.0) | 4 (40.0) | 3 (25.0) | NS |
|  |  | Partner | 9 (9.9) | 2 (4.5) | 1 (4.0) | 3 (30.0) | 3 (25.0) | 0.019 |
| Develop patient feedback data collection instruments to inform audit | 46 (50.5) | Inform | 11 (12.1) | 5 (11.4) | 3 (12.0) | 0 (0.0) | 3 (25.0) | NS |
|  |  | Consult | 20 (22.0) | 7 (15.9) | 6 (24.0) | 2 (20.0) | 5 (41.7) | NS |
|  |  | Involve | 19 (20.9) | 6 (13.6) | 7 (28.0) | 3 (30.0) | 3 (25.0) | NS |
|  |  | Partner | 10 (11.0) | 1 (2.3) | 3 (12.0) | 3 (30.0) | 3 (25.0) | 0.023 |
| Design healthcare staff/professional feedback data collection instruments to inform audit | 28 (30.8) | Inform | 8 (8.8) | 5 (11.4) | 2 (8.0) | 0 (0.0) | 1 (8.3) | NS |
|  |  | Consult | 6 (6.6) | 3 (6.8) | 2 (8.0) | 1 (10.0) | 0 (0.0) | NS |
|  |  | Involve | 10 (11.0) | 4 (9.1) | 3 (12.0) | 3 (30.0) | 0 (0.0) | NS |
|  |  | Partner | 3 (3.3) | 0 (0.0) | 1 (4.0) | 1 (10.0) | 1 (8.3) | NS |
| Plan or conduct interviews or focus groups of patients to inform audit | 42 (46.2) | Inform | 10 (11.0) | 3 (6.8) | 2 (8.0) | 1 (10.0) | 4 (33.3) | NS |
|  |  | Consult | 19 (20.9) | 4 (9.1) | 6 (24.0) | 3 (30.0) | 6 (50.0) | 0.015 |
|  |  | Involve | 18 (19.8) | 5 (11.4) | 6 (24.0) | 4 (40.0) | 3 (25.0) | NS |
|  |  | Partner | 8 (8.8) | 2 (4.5) | 0 (0.0) | 4 (40.0) | 2 (16.7) | 0.001 |
| Plan or conduct interviews or focus groups of healthcare staff/professionals to inform audit | 23 (25.3) | Inform | 5 (5.5) | 2 (4.5) | 0 (0.0) | 0 (0.0) | 3 (25.0) | 0.012 |
|  |  | Consult | 6 (6.6) | 1 (2.3) | 2 (8.0) | 0 (0.0) | 3 (25.0) | 0.033 |
|  |  | Involve | 7 (7.7) | 3 (6.8) | 1 (4.0) | 1 (10.0) | 2 (16.7) | NS |
|  |  | Partner | 4 (4.4) | 1 (2.3) | 0 (0.0) | 1 (10.0) | 2 (16.7) | NS |
| Plan or conduct executive walkabouts | 37 (40.7) | Inform | 13 (14.3) | 8 (18.2) | 2 (8.0) | 0 (0.0) | 3 (25.0) | NS |
|  |  | Consult | 9 (9.9) | 1 (2.3) | 2 (8.0) | 2 (20.0) | 4 (33.3) | 0.009 |
|  |  | Involve | 15 (16.5) | 5 (11.4) | 5 (20.0) | 3 (30.0) | 2 (16.7) | NS |
|  |  | Partner | 7 (7.7) | 2 (4.5) | 2 (8.0) | 1 (10.0) | 2 (16.7) | NS |
| Plan or conduct simulated patient visits | 17 (18.7) | Inform | 4 (4.4) | 2 (4.5) | 1 (4.0) | 0 (0.0) | 1 (8.3) | NS |
|  |  | Consult | 4 (4.4) | 1 (2.3) | 0 (0.0) | 2 (20.0) | 1 (8.3) | 0.049 |
|  |  | Involve | 6 (6.6) | 2 (4.5) | 1 (4.0) | 3 (30.0) | 0 (0.0) | 0.016 |
|  |  | Partner | 1 (1.1) | 1 (2.3) | 0 (0.0) | 0 (0.0) | 0 (0.0) | NS |
| Design or plan specific quality improvement projects | 69 (75.8) | Inform | 20 (22.0) | 9 (20.5) | 4 (16.0) | 1 (10.0) | 6 (50.0) | NS |
|  |  | Consult | 30 (33.0) | 12 (27.3) | 7 (28.0) | 4 (40.0) | 7 (58.3) | NS |
|  |  | Involve | 34 (37.4) | 12 (27.3) | 9 (36.0) | 7 (70.0) | 6 (50.0) | NS |
|  |  | Partner | 17 (18.7) | 5 (11.4) | 4 (16.0) | 4 (40.0) | 4 (33.3) | NS |
| Conduct or execute quality improvement projects | 54 (59.3) | Inform | 15 (16.5) | 7 (15.9) | 3 (12.0) | 1 (10.0) | 4 (33.3) | NS |
|  |  | Consult | 23 (25.3) | 7 (15.9) | 7 (28.0) | 5 (50.0) | 4 (33.3) | NS |
|  |  | Involve | 23 (25.3) | 6 (13.6) | 8 (32.0) | 7 (70.0) | 2 (16.7) | 0.002 |
|  |  | Partner | 14 (15.4) | 2 (4.5) | 3 (12.0) | 5 (50.0) | 4 (33.3) | 0.001 |
| Train staff or health professionals to implement new or improve services | 27 (29.7) | Inform | 7 (7.7) | 4 (9.1) | 1 (4.0) | 1 (10.0) | 1 (8.3) | NS |
|  |  | Consult | 9 (9.9) | 2 (4.5) | 2 (8.0) | 3 (30.0) | 2 (16.7) | NS |
|  |  | Involve | 9 (9.9) | 0 (0.0) | 3 (12.0) | 4 (40.0) | 2 (16.7) | 0.001 |
|  |  | Partner | 7 (7.7) | 0 (0.0) | 1 (4.0) | 3 (30.0) | 3 (25.0) | 0.001 |
| Implement changes emerging from quality improvement projects | 53 (58.2) | Inform | 21 (23.1) | 12 (27.3) | 3 (12.0) | 2 (20.0) | 4 (33.3) | NS |
|  |  | Consult | 20 (22.0) | 6 (13.6) | 6 (24.0) | 3 (30.0) | 5 (41.7) | NS |
|  |  | Involve | 14 (15.4) | 3 (6.8) | 4 (16.0) | 4 (40.0) | 3 (25.0) | 0.046 |
|  |  | Partner | 12 (13.2) | 2 (4.5) | 4 (16.0) | 3 (30.0) | 3 (25.0) | NS |
| Participation in Accreditation preparation or execution | 83 (91.2) | Inform | 26 (28.6) | 12 (27.3) | 7 (28.0) | 1 (10.0) | 6 (50.0) | NS |
|  |  | Consult | 39 (42.9) | 18 (40.9) | 9 (36.0) | 3 (30.0) | 9 (75.0) | NS |
|  |  | Involve | 40 (44.0) | 18 (40.9) | 11 (44.0) | 4 (40.0) | 7 (58.3) | NS |
|  |  | Partner | 22 (24.2) | 8 (18.2) | 9 (36.0) | 2 (20.0) | 3 (25.0) | NS |

NS=not significant
