# Supplementary material for: Hospital capacity for patient engagement in planning and improving health services: a cross-sectional survey
Source: BMC Health Serv Res. 2021 Feb 25;21:179. doi: 10.1186/s12913-021-06174-0 (PMC7908767; doi:10.1186/s12913-021-06174-0)
Supplement: Supplementary file 3 — Additional file 3. PE service delivery by engagement mode hospital type. PE in service delivery activities by engagement mode and hospital type. Table showing summary statistics. [file 12913_2021_6174_MOESM3_ESM.docx]

**Hospital capacity for patient engagement in planning and improving health services: A cross-sectional survey**

Anna R Gagliardi*, Toronto General Hospital Research Institute, University Health Network, Toronto, Canada

Juan Pablo Diaz Martinez, Biostatistics Research Unit, University Health Network, Toronto, Canada

G. Ross Baker, Institute of Health Policy, Management and Evaluation, University of Toronto, Toronto, Canada

Lesley Moody, Princess Margaret Cancer Centre, University Health Network, Toronto, Canada

Kerseri Scane, Patient Partnerships, University Health Network, Toronto, Canada

Robin Urquhart, Department of Community Health and Epidemiology, Dalhousie University, Halifax, Nova Scotia

Walter Wodchis, Institute of Health Policy, Management and Evaluation, University of Toronto, Toronto, Canada

*Corresponding author:

anna.gagliardi@uhnresearch.ca

Toronto General Hospital, 200 Elizabeth Street, 13EN-228, Toronto, Canada, M5G2C4

Additional File 3. PE in service delivery activities by engagement mode and hospital type

| Activity | Overall  n (% of 91) | Mode of engagement | Overall by mode of engagement  n (% of 91) | Engagement mode by hospital type  n (%) | | | | |
| --- | --- | --- | --- | --- | --- | --- | --- | --- |
|  |  |  |  | <100 beds  (n=44) | 100+ beds  (n=25) | Teaching  (n=10) | Specialty  (n=12) | p-value |
| Develop information or communication aids | 84 (92.3) | Inform | 20 (22.0) | 7 (15.9) | 6 (24.0) | 2 (20.0) | 5 (41.7) | NS |
|  |  | Consult | 29 (31.9) | 14 (31.8) | 8 (32.0) | 1 (10.0) | 6 (50.0) | NS |
|  |  | Involve | 42 (46.2) | 15 (34.1) | 13 (52.0) | 7 (70.0) | 7 (58.3) | NS |
|  |  | Partner | 32 (35.2) | 10 (22.7) | 11 (44.0) | 5 (50.0) | 6 (50.0) | NS |
| Develop patient satisfaction or experience data collection instruments | 76 (83.5) | Inform | 22 (24.2) | 9 (20.5) | 4 (16.0) | 2 (20.0) | 7 (58.3) | 0.030 |
|  |  | Consult | 34 (37.4) | 15 (34.1) | 10 (40.0) | 2 (20.0) | 7 (58.3) | NS |
|  |  | Involve | 38 (41.8) | 14 (31.8) | 13 (52.0) | 4 (40.0) | 7 (58.3) | NS |
|  |  | Partner | 23 (25.3) | 7 (15.9) | 8 (32.0) | 3 (30.0) | 5 (41.7) | NS |
| Design clinics and rooms | 67 (73.6) | Inform | 19 (20.9) | 6 (13.6) | 5 (20.0) | 2 (20.0) | 6 (50.0) | NS |
|  |  | Consult | 33 (36.3) | 11 (25.0) | 11 (44.0) | 5 (50.0) | 6 (50.0) | NS |
|  |  | Involve | 32 (35.2) | 13 (29.5) | 10 (40.0) | 6 (60.0) | 3 (25.0) | NS |
|  |  | Partner | 10 (11.0) | 1 (2.3) | 3 (12.0) | 4 (40.0) | 2 (16.7) | 0.006 |
| Design care pathways | 56 (61.5) | Inform | 12 (13.2) | 3 (6.8) | 4 (16.0) | 0 (0.0) | 5 (41.7) | 0.008 |
|  |  | Consult | 27 (29.7) | 10 (22.7) | 8 (32.0) | 2 (20.0) | 7 (58.3) | NS |
|  |  | Involve | 15 (16.5) | 1 (2.3) | 5 (20.0) | 6 (60.0) | 3 (25.0) | <0.001 |
|  |  | Partner | 9 (9.9) | 1 (2.3) | 2 (8.0) | 4 (40.0) | 2 (16.7) | 0.003 |
| Function as patient navigators | 54 (59.3) | Inform | 7 (7.7) | 2 (4.5) | 4 (16.0) | 0 (0.0) | 1 (8.3) | NS |
|  |  | Consult | 11 (12.1) | 3 (6.8) | 4 (16.0) | 0 (0.0) | 4 (33.3) | 0.046 |
|  |  | Involve | 10 (11.0) | 2 (4.5) | 4 (16.0) | 2 (20.0) | 2 (16.7) | NS |
|  |  | Partner | 7 (7.7) | 1 (2.3) | 3 (12.0) | 1 (10.0) | 2 (16.7) | NS |
| Design training programs for patient navigators | 45 (49.5) | Inform | 4 (4.4) | 1 (2.3) | 1 (4.0) | 0 (0.0) | 2 (16.7) | NS |
|  |  | Consult | 9 (9.9) | 2 (4.5) | 3 (12.0) | 0 (0.0) | 4 (33.3) | 0.018 |
|  |  | Involve | 5 (5.5) | 2 (4.5) | 1 (4.0) | 1 (10.0) | 1 (8.3) | NS |
|  |  | Partner | 4 (4.4) | 1 (2.3) | 2 (8.0) | 0 (0.0) | 0 (0.0) | NS |
| Design education programs for patients | 44 (48.4) | Inform | 12 (13.2) | 2 (4.5) | 4 (16.0) | 0 (0.0) | 6 (50.0) | <0.001 |
|  |  | Consult | 28 (30.8) | 4 (9.1) | 10 (40.0) | 6 (60.0) | 8 (66.7) | <0.001 |
|  |  | Involve | 24 (26.4) | 10 (22.7) | 6 (24.0) | 4 (40.0) | 4 (33.3) | NS |
|  |  | Partner | 16 (17.6) | 1 (2.3) | 5 (20.0) | 5 (50.0) | 5 (41.7) | <0.001 |
| Deliver one-on-one or group education to patients | 44 (48.4) | Inform | 10 (11.0) | 1 (2.3) | 4 (16.0) | 0 (0.0) | 5 (41.7) | 0.001 |
|  |  | Consult | 13 (14.3) | 3 (6.8) | 5 (20.0) | 0 (0.0) | 5 (41.7) | 0.009 |
|  |  | Involve | 8 (8.8) | 2 (4.5) | 3 (12.0) | 1 (10.0) | 2 (16.7) | NS |
|  |  | Partner | 12 (13.2) | 0 (0.0) | 5 (20.0) | 3 (30.0) | 4 (33.3) | 0.002 |
| Design the psycho-social or emotional support programs for patients | 38 (41.8) | Inform | 10 (11.0) | 1 (2.3) | 5 (20.0) | 0 (0.0) | 4 (33.3) | 0.005 |
|  |  | Consult | 14 (15.4) | 3 (6.8) | 6 (24.0) | 0 (0.0) | 5 (41.7) | 0.007 |
|  |  | Involve | 10 (11.0) | 2 (4.5) | 3 (12.0) | 2 (20.0) | 3 (25.0) | NS |
|  |  | Partner | 11 (12.1) | 0 (0.0) | 5 (20.0) | 2 (20.0) | 4 (33.3) | 0.004 |
| Deliver one-on-one or group psycho-social or emotional support programs to patients | 34 (37.4) | Inform | 6 (6.6) | 1 (2.3) | 2 (8.0) | 0 (0.0) | 3 (25.0) | 0.033 |
|  |  | Consult | 9 (9.9) | 2 (4.5) | 1 (4.0) | 1 (10.0) | 5 (41.7) | 0.001 |
|  |  | Involve | 7 (7.7) | 1 (2.3) | 2 (8.0) | 1 (10.0) | 3 (25.0) | NS |
|  |  | Partner | 6 (6.6) | 0 (0.0) | 2 (8.0) | 1 (10.0) | 3 (25.0) | 0.019 |
| Deliver presentations about PE programs or PFE program impact to staff, health care professionals and executives | 34 (37.4) | Inform | 10 (11.0) | 3 (6.8) | 2 (8.0) | 0 (0.0) | 5 (41.7) | 0.003 |
|  |  | Consult | 16 (17.6) | 4 (9.1) | 5 (20.0) | 2 (20.0) | 5 (41.7) | NS |
|  |  | Involve | 16 (17.6) | 4 (9.1) | 3 (12.0) | 5 (50.0) | 4 (33.3) | 0.007 |
|  |  | Partner | 20 (22.0) | 5 (11.4) | 6 (24.0) | 4 (40.0) | 5 (41.7) | NS |
| Provide PE orientation or training to staff, health care professionals or executives | 33 (36.3) | Inform | 7 (7.7) | 1 (2.3) | 2 (8.0) | 0 (0.0) | 4 (33.3) | 0.003 |
|  |  | Consult | 14 (15.4) | 3 (6.8) | 2 (8.0) | 3 (30.0) | 6 (50.0) | 0.001 |
|  |  | Involve | 11 (12.1) | 3 (6.8) | 2 (8.0) | 4 (40.0) | 2 (16.7) | 0.028 |
|  |  | Partner | 16 (17.6) | 3 (6.8) | 4 (16.0) | 5 (50.0) | 4 (33.3) | 0.005 |
| Provide PE orientation or training to new PFAC members | 29 (31.9) | Inform | 12 (13.2) | 4 (9.1) | 2 (8.0) | 1 (10.0) | 5 (41.7) | 0.020 |
|  |  | Consult | 22 (24.2) | 6 (13.6) | 7 (28.0) | 1 (10.0) | 8 (66.7) | 0.001 |
|  |  | Involve | 18 (19.8) | 9 (20.5) | 3 (12.0) | 2 (20.0) | 4 (33.3) | NS |
|  |  | Partner | 23 (25.3) | 6 (13.6) | 6 (24.0) | 5 (50.0) | 6 (50.0) | 0.016 |
| Deliver presentations about PE activities or impact to external audiences | 25 (27.5) | Inform | 8 (8.8) | 3 (6.8) | 3 (12.0) | 0 (0.0) | 2 (16.7) | NS |
|  |  | Consult | 12 (13.2) | 3 (6.8) | 4 (16.0) | 2 (20.0) | 3 (25.0) | NS |
|  |  | Involve | 17 (18.7) | 6 (13.6) | 2 (8.0) | 6 (60.0) | 3 (25.0) | 0.003 |
|  |  | Partner | 23 (25.3) | 8 (18.2) | 6 (24.0) | 4 (40.0) | 5 (41.7) | NS |

NS=not significant
