# Supplementary material for: Hospital capacity for patient engagement in planning and improving health services: a cross-sectional survey
Source: BMC Health Serv Res. 2021 Feb 25;21:179. doi: 10.1186/s12913-021-06174-0 (PMC7908767; doi:10.1186/s12913-021-06174-0)

**Hospital capacity for patient engagement in planning and improving health services: A cross-sectional survey**

Anna R Gagliardi*, Toronto General Hospital Research Institute, University Health Network, Toronto, Canada

Juan Pablo Diaz Martinez, Biostatistics Research Unit, University Health Network, Toronto, Canada

G. Ross Baker, Institute of Health Policy, Management and Evaluation, University of Toronto, Toronto, Canada

Lesley Moody, Princess Margaret Cancer Centre, University Health Network, Toronto, Canada

Kerseri Scane, Patient Partnerships, University Health Network, Toronto, Canada

Robin Urquhart, Department of Community Health and Epidemiology, Dalhousie University, Halifax, Nova Scotia

Walter Wodchis, Institute of Health Policy, Management and Evaluation, University of Toronto, Toronto, Canada

*Corresponding author:

anna.gagliardi@uhnresearch.ca

Toronto General Hospital, 200 Elizabeth Street, 13EN-228, Toronto, Canada, M5G2C4

Additional File 4. Association of organizational capacity for PE and quality measures

**Hand-washing**


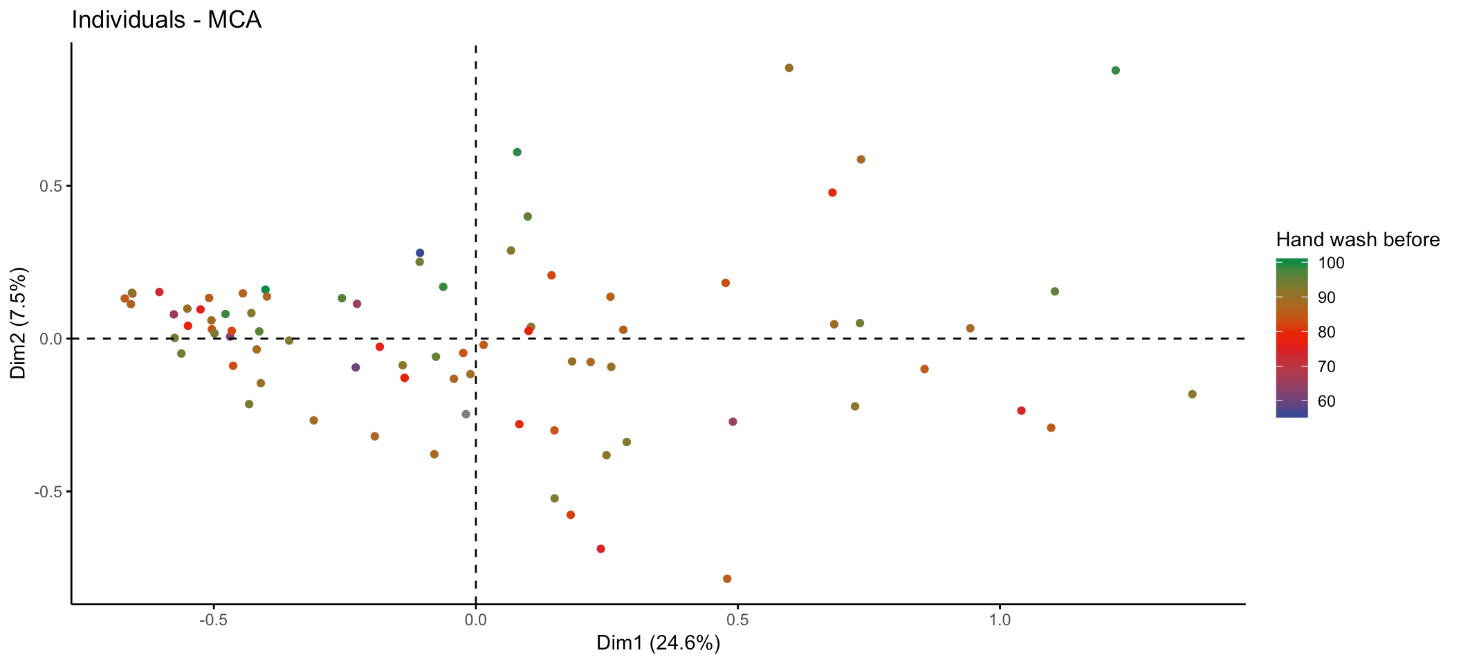


**Infection rate**


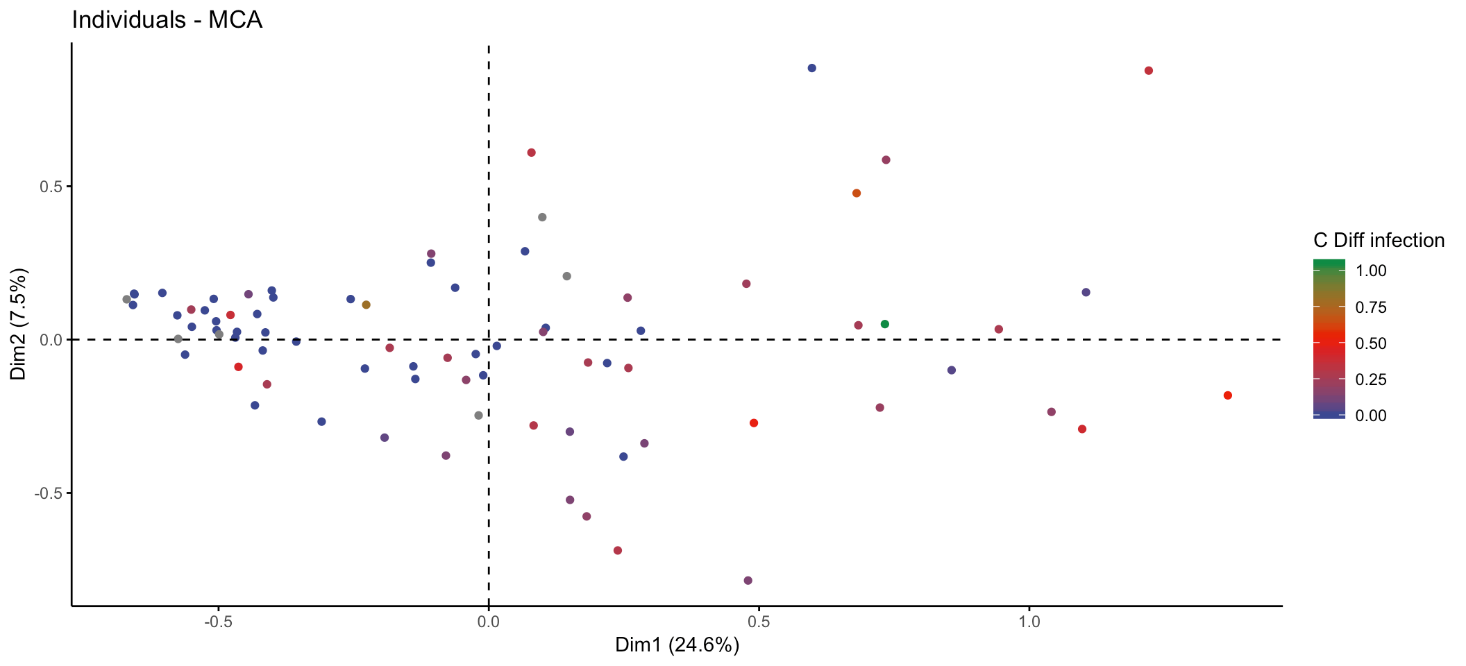


**Readmission rate**


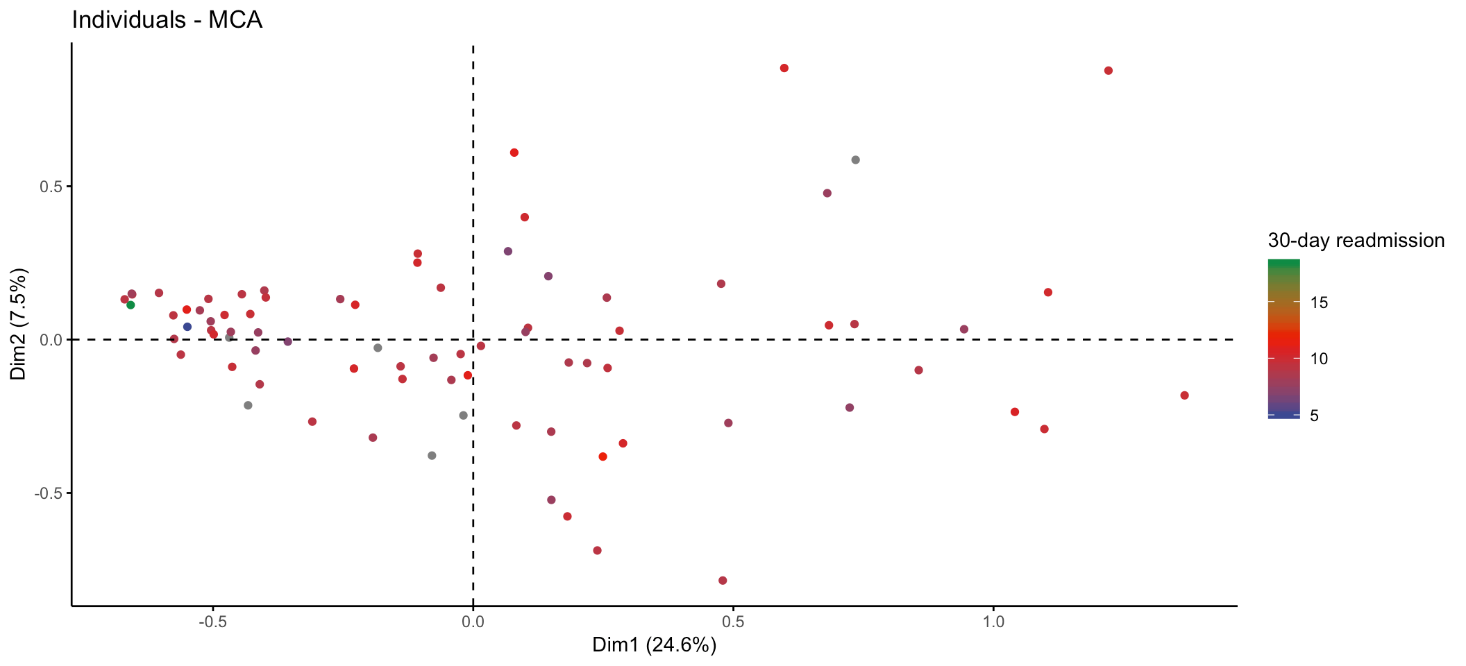

Supplement: Supplementary file 4 — Additional file 4. PE capacity and quality measures. Association of organizational capacity for PE and quality measures. Multiple correspondence analysis grids. [file 12913_2021_6174_MOESM4_ESM.docx]
